# Interlaminar Fracture Behavior of Carbon Fiber/Polyimide Composites Toughened by Interleaving Thermoplastic Polyimide Fiber Veils

**DOI:** 10.3390/ma14102695

**Published:** 2021-05-20

**Authors:** Bangwei Lan, Yi Liu, Song Mo, Minhui He, Lei Zhai, Lin Fan

**Affiliations:** 1 Key Laboratory of Science and Technology on High-Tech Polymer Materials, Institute of Chemistry, Chinese Academy of Sciences, Beijing 100190, China; lanbangwei@iccas.ac.cn (B.L.); mosong@iccas.ac.cn (S.M.); heminhui@iccas.ac.cn (M.H.); zhailei@iccas.ac.cn (L.Z.); 2 School of Chemical Sciences, University of Chinese Academy of Sciences, Beijing 100049, China

**Keywords:** polyimide, composites, interlaminar fracture toughness, interleaved laminates

## Abstract

Carbon fiber reinforced thermosetting polyimide (CF/TSPI) composites were interleaved with thermally stable thermoplastic polyimide (TPPI) fiber veils in order to improve the interlaminar fracture toughness without sacrificing the heat resistance. Both of the mode I and mode II interlaminar fracture toughness (G_IC_ and G_IIC_) for the untoughened laminate and TPPI fiber veils interleaved laminates were characterized by the double cantilever beam (DCB) test and end notch flexure (ENF) test, respectively. It is found that the TPPI fiber veils interleaved laminates exhibit extremely increased fracture toughness than the untoughened one. Moreover, the areal density of TPPI greatly affected the fracture toughness of laminates. A maximum improvement up to 179% and 132% on G_IC_ and G_IIC_ is obtained for 15 gsm fiber veils interleaved laminate, which contributes to the existence of bicontinuous TPPI/TSPI structure in the interlayer according to the fractography analysis. The interlaminar fracture behavior at elevated temperatures for 15 gsm fiber veils interleaved laminate were also investigated. The results indicated that the introduction of thermally stable TPPI fiber veils could enhance the fracture toughness of CF/TSPI composites by exceeding 200% as compared to the untoughened one even as tested at 250 °C.

## 1. Introduction

Carbon fiber reinforced thermosetting polyimide matrix composites (CF/TSPI) have been widely used in aviation and aerospace structural applications owing to the combination of excellent heat and radiation resistance, high specific modulus and strength, as well as high dimensional stability [1,2]. The hot zones of the aircraft, such as engine components, are often fabricated from CF/TSPI composites because they can offer better thermal stability than most of other polymeric matrix composites [3]. However, the traditional high temperature resistant thermosetting polyimide matrix resin usually has a rigid backbone and/or high crosslinking density, which leads to the CF/TSPI composites with insufficient fracture toughness and impact resistance. Delamination failure is prone to happen in the CF/TSPI structural composites during their service lives, which seriously affects the safety of the aircraft systems.

Many methods have been developed to improve interlaminar fracture toughness and delamination resistance of CF/TSPI composites over recent years. The most direct strategy is matrix toughening, which includes chemical modification and physical blending toughening. However, chemical structure modification of the polymeric matrix for toughening always results in the sacrifice of other properties of composites [4]. The improvement in toughness of composites is usually accompanied by the decrease of the glass transition temperature (T_g_) and modulus [5,6]. For physical blending toughening, high molecular weight thermoplastic polymers [7,8] or reactive rubbers [9] are introduced in the thermosetting polymeric matrix, which inevitably results in a great increase in melt viscosity of the toughened matrix. In the subsequent impregnating and curing process, the melt flow of the matrix resin is difficult to carry out, leading to composites with high porosity.

Recently, interleaving has been proposed to toughen the interlayer region of composites with thermoplastic polymers (TP), rubbers [10], or carbon nanotubes reinforced resins [11]. Among all the interleaving methods, TP interleaving is considered as a potential method to improve the interlaminar fracture toughness of composites without compromising other properties [12]. The basic thought of this technique is selectively inserting tough TP layers into the weak interlaminar region of composites. The material patterns of TP for interleaving mainly include powder [13], microparticle [14,15], film [16,17], and fiber [18]. Liu et al. [19] used TP powder and film to interleave CF/TSPI composites for improving the mode I interlaminar fracture toughness. However, the TP powders could not disperse homogenously in the interlayers due to migration caused by resin flow; on the other hand, the resin flow through the thickness direction would be impeded by the continuous film during the curing of TP film interleaved CF/TSPI composites. Therefore, the toughness enhancement of the corresponding composites was limited. The maximum improvement of G_IC_ was only 36% and 48% for powder and film interleaved composites, respectively. Comparing this with TP powder or film interleaving, interleaving composites with electrospun TP fiber veils has been proved to be a more effective method to enhance the interlaminar fracture toughness of composites [20,21]. Electrospun TP fiber veils have the advantages of being self-supporting, high porosity, and having a large surface area-to-volume ratio [22]. As a consequence, they can be uniformly placed between reinforcing plies, meanwhile their porous nature is beneficial to resin flow in the curing process. Li et al. investigated the effect of interleaving material patterns on the toughness of corresponding interleaved composites [23]. Carbon fiber/epoxy composites (CF/EP) were separately interleaved with electrospun polysulfone (PSF) fiber and PSF film in their research. The results indicated that 281% improvement in G_IC_ properties was obtained in PSF fiber interleaved composites, while only 100% improvement in G_IC_ properties was obtained for PSF film interleaved composites. Zhang et al. [24] reported the toughening mechanism of TP fibers interleaving by analyzing the micro-morphologies of poly(e-caprolactone) (PCL) fibers interleaved CF/EP composites. They suggested that the curing reaction-induced phase separation of PCL happened, and the ductile TP-rich particulate microphases were formed on the delamination plane. These particulate phases could serve as sites to initiate shear bands for forming plastic deformation zones, which improved the toughness of composites.

In spite of electrospun TP fiber veils interleaving achieving a lot of successful applications in CF/EP composites, to the best of our knowledge, the research focused on TP fiber veils interleaved CF/TSPI composites is still very limited up to now. It is well known that the curing temperature of CF/TSPI composite is generally 320–370 °C, which makes the conventional TP fiber veils used in interleaved CF/EP composites unsuitable for interleaving CF/TSPI composites due to the poor compatibility of the TP and TSPI matrix. Moreover, the much lower heat resistance of conventional TP would reduce the high-temperature performance of interleaved CF/TSPI composites. Thermoplastic polyimide (TPPI) is a kind of linear polymer with high molecular weight, which has similar chemical structure and good miscibility with TSPI, as well as the advantages of high temperature resistance and excellent toughness [25,26]. Therefore, TPPI fiber veils are considered as an ideal interleaving material to toughen the CF/TSPI composites. However, almost no related research has been reported in the literature. The effectiveness and toughening mechanism of this kind of interleaving material are still unknown. Furthermore, the interlaminar fracture behavior of TP interleaved CF/TSPI composites at elevated temperature is rarely investigated, which is critical for high temperature applications. Therefore, a comprehensive and systematic study is necessary to disclose the fracture behavior of TPPI fiber veils interleaved CF/TSPI composites under various temperatures.

In this research, the thermally stable TPPI fiber veils were fabricated by electrospinning and applied to toughen the CF/TSPI composite by interleaving. In consideration of the fracture behavior significantly affected by the ratio of TPPI/TSPI in the interlayer, the correlation of area density of TPPI fiber veils with the mode I and mode II fracture toughness of interleaved laminates were investigated and the mentioned properties were compared with the untoughened one. The characteristic of fracture surface and cracks development and the toughening mechanism for the interleaved and untoughened laminates were discussed. In addition, as an effort to further disclose the fracture behavior of interleaved composites at an elevated temperature, mode I and mode II fracture toughness of these laminates were tested at 200 and 250 °C; meanwhile, their corresponding fracture morphologies were investigated.

## 2. Materials and Methods

### 2.1. Materials

The dianhydride and anhydride monomers, i.e., 2,3,3’,4’-biphenyltetracarboxylic dianhydride (a-BPDA), 3,3′,4,4′-benzophenonetetracarboxylic dianhydride (BTDA), 4-phenylethynyl phthalic anhydride (PEPA), and phthalic anhydride (PA), were purchased from Changzhou Sunlight Pharmaceutical Co., Changzhou, China. BTDA and a-BPDA were dried in a vacuum oven at 160 °C for 8 h before use. PEPA and PA were dried, respectively, at 120 and 80 °C for 8 h under vacuum prior to use. The diamines of 4,4′-oxydiaminine (4,4′-ODA) and 5-amino-1-(4-aminophenyl)-1,3,3-trimethylindane (DAPI) were obtained from Shandong Guansen Co., Shandong, China and Changzhou Sunlight Pharmaceutical Co., Changzhou, China, respectively. All the diamines were used directly without further purification. Anhydrous ethanol and *N*,*N*′-dimethylacetamide (DMAc) were supplied by Beijing Chemical Works, Beijing, China, and used without further purification. The commercially available T800H carbon fibers were used as received.

### 2.2. Preparation of Unidirectional CF/TSPI Prepreg

A precursor solution of thermosetting polyimide oligomer with a solid content of 45 wt.% in the mixture solvents of ethanol and DMAc was prepared from 4,4′-ODA, diethyl ester of 2,3,3’,4’-biphenyltetracarboxylic acid (a-BPDE), and monoethyl ester of 4-phenylethynylphthalic acid (PEPE) by the in-situ polymerization of monomer reactants (PMR) process according to the method previously reported [6,27,28,29,30]. The number average molecular weight (M_n_) of the thermosetting polyimide oligomer is around 4500 g/mol.

**Figure 1:**
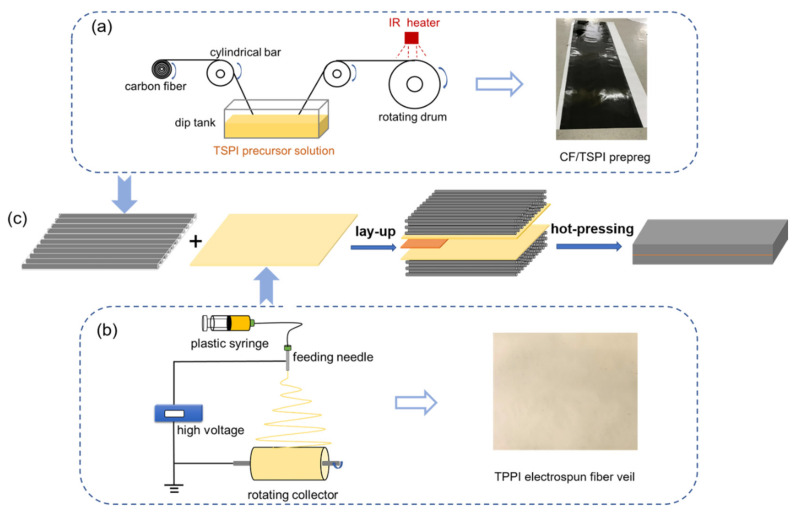
The process schematic of (**a**) preparation of unidirectional CF/TSPI prepreg; (**b**) electrospinning of TPPI fiber veil; (**c**) preparation of TPPI fiber veils interleaved CF/TSPI laminates.

The unidirectional CF/TSPI prepreg was fabricated by impregnation of T800H carbon fibers with the precursor solution of thermosetting polyimide oligomer as illustrated in Figure 1a. In this process, an automatic impregnation machine (Xi’an Long Technology & Development Co., Xi’an, China) was used, pulling the continuous T800H carbon fiber through a dip tank containing the precursor solution. The fiber was fed through a series of cylindrical bars and immersed in the solution to promote fiber wetting. The prepreg was picked up with a rotating drum and dried by an IR heater at 60 °C for 12 h to remove most of ethanol solvent during the winding process. The prepreg was subsequently removed from the drum and cut into desirable sizes. Finally, the CF/TSPI prepreg was obtained after further drying in a vacuum oven at 180 °C for 2 h to remove residual solvents. The prepreg contained around 40 wt.% of polyimide matrix resin.

### 2.3. Preparation of Electrospun Thermoplastic Polyimide Fiber Veils

The poly (amid acid) precursor of thermoplastic polyimide was synthesized by solution polycondensation of BTDA and DAPI in DMAc. PA was used as an end-cap to control the molecular weight. The PAA precursor was fully converted into polyimide by the chemical imidization method [31,32]. The TPPI resin powder was obtained after a precipitation and drying process. The number average molecular weight of TPPI resin determined by gel permeation chromatography (GPC) is around 30,000 g/mol.

The electrospinning process was carried out in a single-nozzle electrospinning system (TL-01, Tongli nano-technology Co., Shenzhen, China) as presented in Figure 1b. The TPPI resin powders were dissolved in DMAc and stirred for 12 h to obtain a homogeneous solution with the solid content of 20 wt.%. Then, the TPPI solution was put into a 10 mL plastic syringe connected to the feeding needle by plastic pipes. The needle was placed in a closed electrostatic chamber where temperature and humidity can be controlled. The TPPI solution was injected by a pump (LSP02-2B, Longer Precision Pump Co., Baoding, China) with the flow rate of 0.7 mL/h, and spun at an applied voltage of 16 kV. TPPI fibers were collected by aluminum foil, which was bonded on the rotating collector with a rotary speed of 500 rpm. The distance between the needle tip and the collector was 20 cm. The fiber veils can easily peel off from the aluminum foil without damages. The residue organic solvent in the fiber veils was removed after drying in a vacuum oven at 180 °C for 2 h. The areal density and thickness of fiber veils can be controlled by changing the spinning time. The fiber veils with the areal density of 5, 10, 15, and 20 gsm (grams per square meter), respectively, were prepared by the above method in this study.

### 2.4. Preparation of TPPI Fiber Veils Interleaved Composites

The TPPI fiber veils interleaved CF/TSPI laminates, for investigating the interlaminar fracture behavior, were prepared by the procedure shown in Figure 1c. The 20 piles of unidirectional CF/TSPI prepreg with the size of 20 × 20 cm were laid in 0° orientation, and two fiber veils were placed into the mid-plane (between the 10th and 11th plies) of the prepreg. For inducing the initial delamination, a Kapton film with a thickness of 25 μm was inserted between two fiber veils. The as-received laminate was put into a vacuum oven at 200 °C for 30 min to perform degassing and consolidation. Then, the laminate was placed in a hot press and thermally cured at 370 °C for 2 h under 2.5 MPa pressure. A series of laminates interleaved with TPPI fiber veils of different areal densities were fabricated to explore the effect of the interleaving fiber content on the interlaminar fracture toughness.

### 2.5. Characterization

#### 2.5.1. Mode I and Mode II Interlaminar Fracture Properties

**Figure 2:**
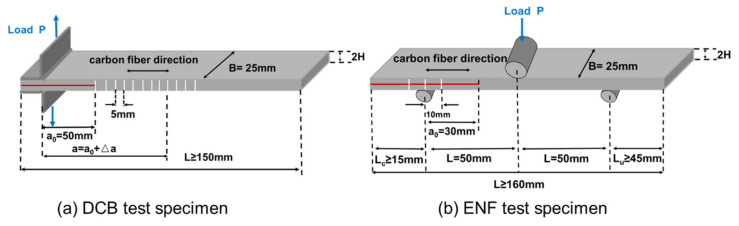
The geometry schematic of DCB (**a**) and ENF (**b**) test specimens.

Mode I interlaminar fracture toughness was characterized by the double cantilever beam (DCB) test in accordance with ASTM D5528-2013. Figure 2a shows the geometric dimension of DCB specimens. Two piano hinges were bonded on both sides of the pre-cracked end of each specimen using a polyimide adhesive, and the initial delamination length was 50 mm. To observe the crack growth, the edges of specimens were coated with white correction fluid and symbol lines were draw on the coating. The break of symbol lines can locate the position of crack tips during crack propagation. The DCB measurements were carried out in an Instron 5567 universal tester at a cross-head speed of 2 mm/min (for high temperature DCB test, the heat chamber with a window was used). The G_I_ was calculated by the Equation (1) according to the modified beam theory method.

(1) $$G_{I}=\frac{3P\delta }{2b\left(a_{0}+\Delta a\right)}\;$$

where P is fracture load, δ is load point displacement, b is specimen width, a_0_ is initial delamination length, and Δa is crack propagation length. The mode I initiation energy release rate (G_IC_) is determined using the data at the point of deviation from linearity in the load–displacement curve. The mode I propagation energy release rate (G_IR_) is the average value of G_I_ when the crack propagation is relatively stable (Δa > 10 mm) [33]. It is worth mentioning that the loading speed has an important impact on the mode I fracture toughness. According to the research by Liu [34] and May [35], the DCB specimen tends to open asymmetrically when the high loading speed is applied, which results in a mixed-mode fracture. Moreover, it is a challenge to measure forces and crack growth at high rates of loading, which leads to the difficulty of recording the R-curve.

Mode II interlaminar fracture toughness was characterized by the end notch flexure (ENF) test in accordance with ASTM D7905M-2014. Figure 2b shows the geometric dimension of ENF specimens. The ENF measurements were carried out in an Instron 5567 universal tester at a cross-head speed of 0.5 mm/min (for high-temperature ENF test, the heat chamber with a window was used). G_IIC_ was calculated by Equation (2)

(2) $$G_{\mathrm{IIC}}=\frac{{3\mathrm{mP}}_{\max}{}^{2}a_{0}{}^{2}}{2B}\;$$

where m is the compliance calibration coefficient, which is calculated according to the reference [36], P_max_ is the maximum force from the fracture test, a_0_ is the initial crack length used in the fracture test (30 mm), and B is specimen width.

#### 2.5.2. Morphology Observation

Scanning electron microscopy (SEM) was recorded on a Hitachi SU8020 instrument (Tokyo, Japan) to investigate the diameters and morphology of TPPI fibers as well as delamination fracture surface of composites. The samples were coated with Pt in a sputter coater before SEM measurement. The diameter of TPPI fibers was determined by SEM micrograph with the commercial image software Image Pro Plus 6.0 (Media Cybernetics, Rockville, MD, USA) and calculated at least 50 fibers as the average diameter.

#### 2.5.3. Thermal Properties

Differential scanning calorimetry (DSC) and thermogravimetric analysis (TGA) of TPPI fiber veils were carried on a TA Q100 instrument at a heating rate of 5 °C/min in nitrogen and a TA Q50 instrument (TA Instrument, Newcastle, DE, USA) at a heating rate of 10 °C/min in air, respectively.

## 3. Results

### 3.1. Characterization of Electrospun TPPI Fiber Veils

**Figure 3:**
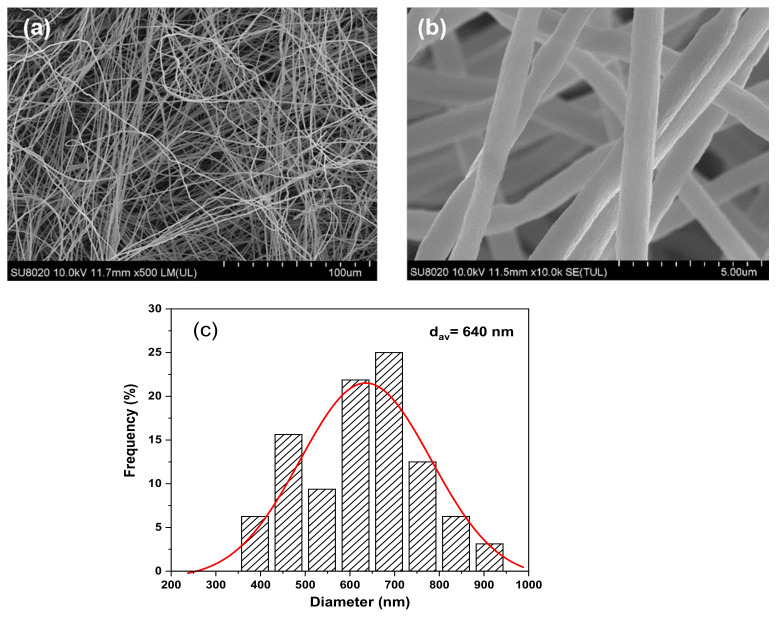
SEM micrographs of TPPI fiber veils in low resolution (**a**) and high resolution (**b**), and the calculated fiber diameters distribution (**c**).

The morphology of TPPI fiber veils was investigated by SEM micrographs and shown in Figure 3a,b. It is obvious that the fiber veils are randomly oriented and form the heterogeneous crossing junctions with beads-free morphology. There are lots of cavities between the crossing fibers could be observed. The statistical graph of fiber diameters determined by SEM micrograph is illustrated in Figure 3c. The diameters of the fibers are in the range from 350 nm to 950 nm, and the average diameter is 640 nm.

**Figure 4:**
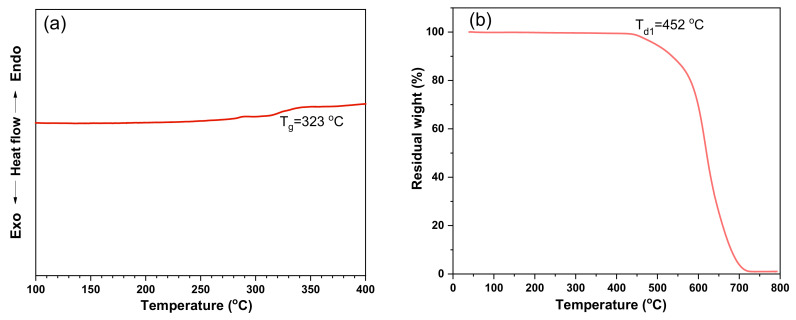
DSC curve (**a**) and TGA curve (**b**) of TPPI fiber veils.

The thermal properties of TPPI fiber veils are also detected by differential scanning calorimetry (DSC) and thermogravimetry analysis (TGA) and the results are shown in Figure 4. The glass transition temperature (T_g_) of TPPI fiber veils determined by the DSC curve is 323 °C, which is not significantly different to that of the cured TSPI matrix (328 °C). The high T_g_ of TPPI fiber veils could ensure that the composites, after toughening with interleaved thermoplastic polyimide, do not sacrifice their heat resistance. Moreover, the TPPI fiber veils exhibit that the decomposition temperature at 1% of weight loss (T_d1_) is 452 °C as tested by TGA. It is well known that the thermosetting polyimide oligomers end-capped with PEPA are generally cured at 370 °C. The high thermal stability of TPPI fiber veils could guarantee it without degradation in the composite curing process.

### 3.2. Mode I and Mode II Fracture Toughness of Laminates

**Figure 5:**
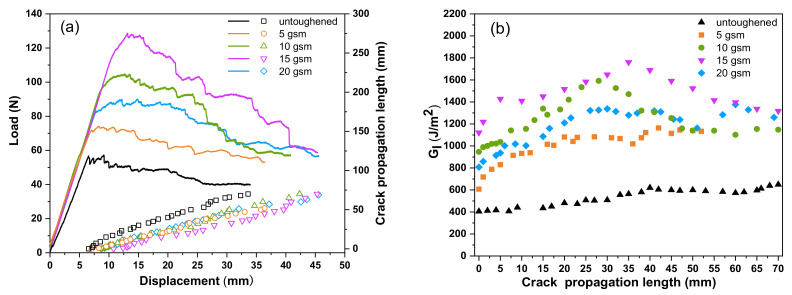
DCB test results of untoughened laminate and the laminates interleaved with different areal densities of TPPI fiber veils: (**a**) Load–displacement curves and crack propagation length–displacement curves, as well as (**b**) the corresponding R-curves.

The fracture toughness of CF/TSPI laminates interleaved with TPPI fiber veils was characterized by mode I and mode II loading via double cantilever beam (DCB) and end notch flexure (ENF) tests, respectively. The DCB test results of untoughened laminate and the laminates interleaved with different areal densities of TPPI fiber veils are shown in Figure 5. It is found that all load-displacement curves exhibit a similar shape, in which the load force increases linearly with the load displacement increasing until crack initiation (Figure 5a). Then, the load force starts to decline and fluctuate because of the crack propagation. All interleaved laminates reveal the delaying of crack initiation and exhibit the dramatic enhancement of maximum load forces as compared with the untoughened specimens. It is suggested that the interleaved laminates have relatively higher G_IC_ value than the untoughened one. The maximum load obtained for the 15 gsm TPPI fiber veils interleaved laminate is around 128 N, which is almost 2.5 times higher than that of the untoughened laminate. Furthermore, the interleaved laminates reveal the higher load force in the fluctuation stage accompanied with the smaller slope of crack propagation length-displacement curves, which demonstrated a higher fracture energy dissipation rate and slower crack propagation. Therefore, it indicates that the G_IR_ values are significantly enhanced [37]. The R-curves of these laminates, which indicate the relationship between the G_I_ and the crack propagation length, are displayed in Figure 5b. The first point in the R-curves represents the initiation of the interlaminar fracture. The interleaved laminates give the relatively higher G_I_ values in the initiation stage (G_IC_) as compared with the untoughened one. Moreover, the interleaved laminates also have higher G_I_ values in the propagation stage (G_IR_) than the untoughened one.

The G_IC_ and G_IR_ values of these laminates are summarized in Table 1. The G_IC_ and G_IR_ values for the untoughened laminate are 418 and 602 J/m^2^, respectively. For the interleaved laminates, both of the G_IC_ and G_IR_ values exhibit an increasing trend followed by decreasing with the rising of areal densities of TPPI veils. The introduction of 5 gsm TPPI fiber veils in the interlayer can lead to an increase of G_IC_ to 671 J/m^2^ and G_IR_ to 928 J/m^2^, respectively. The laminate interleaved with 15 gsm TPPI fiber veils displays the significant improvement of mode I fracture toughness, which give the highest G_IC_ and G_IR_ values of 1167 and 1542 J/m^2^, respectively. The corresponding G_IC_ and G_IR_ values increased by 179% and 156% compared to the untoughened one, respectively. Similar tendency was also detected in other researches. Heijden et al. [38] found that the G_IC_ of electrospun PCL nanofiber interleaved epoxy composites increases rapidly with increase of nanofiber areal density, as the extent of plastic deformation in the epoxy/PCL interlayer will be significantly enhanced with the increase of the ductile PCL component. However, for the 20 gsm TPPI fiber veils interleaved laminate, the G_IC_ and G_IR_ values declined to 733 and 1265 J/m^2^. That means there is an optimal areal density of interleaved TPPI, above which the enhancement effect of G_IC_ begins to decline. This phenomenon may be related to the difference in the crack propagation mechanism as more TPPI fiber veils are interleaved in the composites, which will be discussed in detail in Section 3.3.

**Figure 6:**
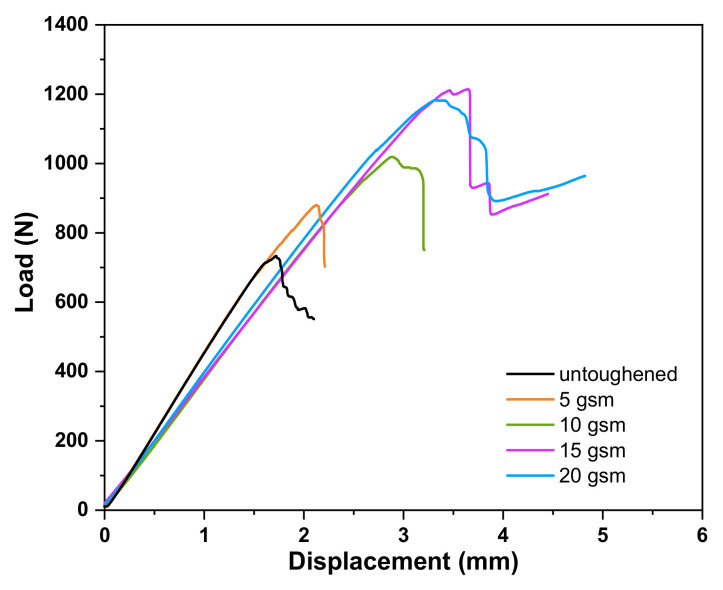
Load–displacement curves in ENF tests of untoughened laminate and the laminates interleaved with different areal densities of TPPI fiber veils.

ENF tests were performed to investigate the impact of TPPI fiber veils on the mode II fracture toughness of these laminates. Figure 6 shows the mode II load–displacement curves of the untoughened laminate and the laminates interleaved with different areal densities of TPPI fiber veils. In all load–displacement curves, the load force enhances linearly with the increasing of displacement until the delamination occurred, then a sudden drop in load force can be observed due to the crack propagation. The maximum load force in load–displacement curves of all interleaved laminates is higher than that of the untoughened one, which indicates that the interleaved laminates required higher energy for crack propagation. The values of G_IIC_ of these laminates are also listed in Table 1. The 15 gsm TPPI fiber veils interleaved laminate also provides the best mode II fracture toughness property, which give the G_IIC_ value of 2042 J/m^2^ and increases by 132% compared to that of the untoughened laminate. Moreover, the relationship between G_IIC_ and areal density of TPPI fiber veils interleaved in the laminates shows similar results as those of the DCB test.

The results of our work indicated that the thermoplastic polyimide fiber veils are more effective in enhancing the fracture toughness of polyimide composites compared with films and powders. According to the study of Liu et al. [19], the mode I and mode II fracture toughness of polyimide composites toughened with TPPI films and powders can only reach 459 and 1100 J/m^2^. In our work, the composite interleaved with TPPI fiber veils displays G_IC_ and G_IIC_ values as high as 1167 and 2042 J/m^2^, which are 2.54 and 1.86 times of the formers, respectively. This is because fiber veils can be uniformly placed between reinforcing plies, and their porous structure is beneficial to resin flow in the curing process. Therefore, it can be concluded that interleaving the thermoplastic polyimide fiber veils into the polyimide composites can significantly enhance the interlaminar fracture toughness.

### 3.3. Fracture Morphology Analysis

**Figure 7:**
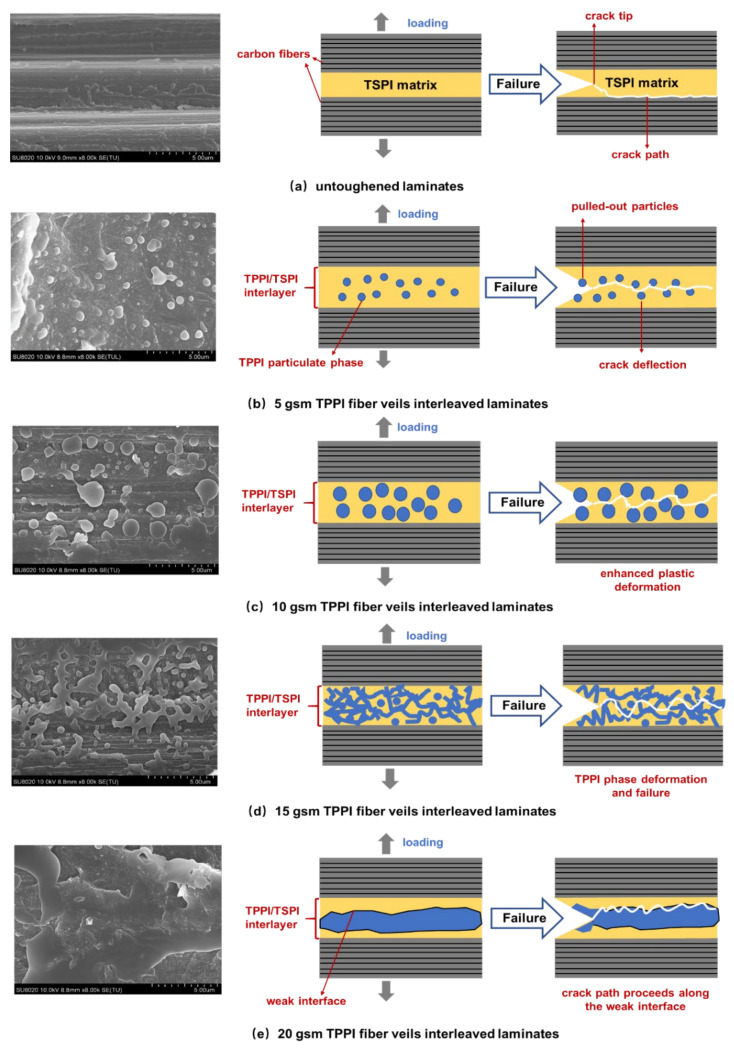
SEM micrographs of mode I fracture surfaces of untoughened and interleaved laminates and the schematic diagrams of the illustrated interlayer structure. (**a**) untoughened laminates; (**b**) 5 gsm TPPI fiber veils interleaved laminates; (**c**) 10 gsm TPPI fiber veils interleaved laminates; (**d**) 15 gsm TPPI fiber veils interleaved laminates; (**e**) 20 gsm TPPI fiber veils interleaved laminates.

The mode I and mode II fracture surfaces of the untoughened and interleaved laminates were investigated to clarify the difference of toughening mechanism. The SEM micrographs of mode I fracture surfaces of these laminates and the schematic diagrams of the corresponding interlayer structure are illustrated in Figure 7. It can be seen that the fracture surface of untoughened laminate is quite smooth and the carbon fibers are exposed in the fracture surface without covered resin (Figure 7a), which is associated with a typical brittle fracture induced by the debonding of carbon fiber/matrix resin interface. It is indicated that the mode I fracture toughness for the untoughened laminate mainly depends on the adhesive strength between the carbon fiber and TSPI matrix (adhesive failure), as a result, the crack propagation path proceeds along the interface.

As a comparison, the interleaved laminates display the relatively rough fracture surfaces with the TPPI and TSPI phase separation morphology. This can be explained by the TPPI fiber veils melted and mixed with TSPI oligomer during the initial curing procedure followed by the excluding from the crosslinked TSPI. For the 5 gsm fiber veils interleaved laminate, the TPPI spherical-like particles isolated dispersion in a continuous TSPI matrix is observed in the fracture surface. The TPPI particles could induce the cracks deflection and result in energy dissipation leading to an improvement in toughness. Moreover, it is found that these TPPI particles are pulled out from the TSPI matrix during the DCB test, which also leads to the toughness increasing because more energy could be absorbed [39].

As the areal density of fiber veils increased to 10 gsm, more TPPI particles with a larger diameter were detected on the fracture surface. The enhancement of the plastic deformation caused by more TPPI components in the interlayer may be another reason for the improvement of mode I fracture toughness. Cheng et al. [40] also found that the plastic yield zone radius in the interlayer of PES fiber veils interleaved composites grew with the increase in the PES microspheres formed in the interlayer. For the 15 gsm fiber veils interleaved laminate, a completely different phase structure was observed in the mode I fracture surface (Figure 7d). It is indicated that a bicontinuous phase structure of TPPI and TSPI accompanied by a TPPI particulate phase is formed in the interlayer [41]. The interpenetrated TPPI/TSPI structure greatly enhances the ductility of the interlayer. When the crack passes through this layer (cohesive failure), the plastic deformation and failure of the TPPI phase would absorb a large quantity of energy, which would significantly improve the mode I fracture toughness of specimen.

When the areal density of interleaving fiber veils increased to 20 gsm, a continuous TPPI-rich phase is formed in the interlayer. In this situation, the content of TPPI in the interlayer greatly exceeded that of TSPI, and the TPPI component would agglomerate in the mid-plane due to its much higher melt viscosity than the TSPI component. As detected in Figure 7e, a weak interface between the continuous TPPI phase and the TSPI matrix is formed, in which the crack can easily pass through. It resulted in the decline of fracture toughness for the 20 gsm fiber veils interleaved laminate. The crack propagation mechanism of mode I fracture surfaces for these laminates can explain the difference in G_IC_ and G_IR_ as mentioned above.

**Figure 8:**
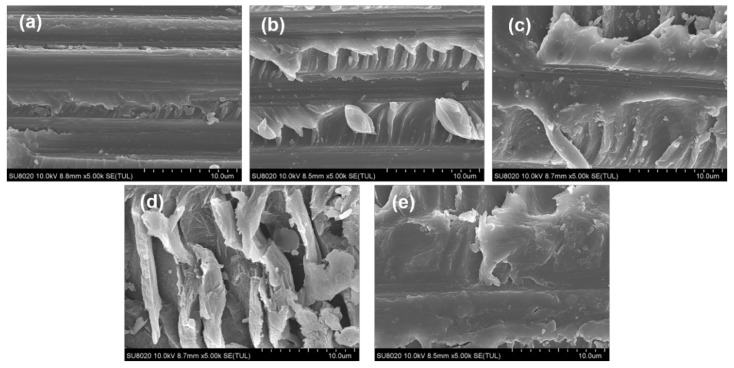
SEM micrographs of mode II fracture surfaces for laminates with different areal densities of interleaves: (**a**) Untoughened, (**b**) 5 gsm, (**c**) 10 gsm, (**d**) 15 gsm, (**e**) 20 gsm.

The fracture behavior of the untoughened and interleaved laminates under the mode II load were also investigated by the fracture surface after ENF tests and presented in Figure 8. It is obvious that a typical hackle-like resin fragments morphology can be observed in all specimens, which is induced by mode II shear stress and microcrack propagation [42]. The crack growth paths under the mode II load are more complicated than under the mode I load, hence the phase structure of TPPI is difficult to be identified in the fracture surface. The untoughened laminate shows the relatively smooth fracture surface and gives a shallow corrugated hackles, which is related to the brittleness of TSPI matrix resin and low adhesive strength of the interface between the carbon fiber and the matrix resin. That means the failure mechanism for the untoughened specimen is dominated by hackle formation and interfacial failure. For the interleaved laminates, a type of banded structure can be observed on the fracture surface, which is induced by regular interlaminar crack crossing. The crossing means the crack is deflected away from one side of the interlayer to the other [43]. Moreover, the fracture surfaces of interleaved laminates reveal denser banded structures when the areal density of interleaving fiber veils increases from 5 to 15 gsm. It is implied that the extent of interlaminar crack crossing is improved due to the intensification of crack deflection. Therefore, more fracture energy will be dissipated due to the extension of crack. However, banded morphology was disappeared in the fracture surface of 20 gsm fiber veils interleaved laminate. It is found that the carbon fibers are covered by TPPI to form a weak interface; therefore, the interlaminar crack crossing is hindered and the mode II toughness tends to decline. The results deduced from mode II fracture surfaces are consistent with those for the mode I fracture surfaces.

### 3.4. Interlaminar Fracture Behavior at Elevated Temperature

The polyimide composites would inevitably be exposed to elevated temperature environments when used in aviation and aerospace applications. The interlaminar fracture behavior of untoughened and fiber veils interleaved laminates at different temperature was investigated. The 15 gsm fiber veils interleaved laminate, which exhibited the highest fracture toughness at room temperature compared to the others, was selected as the representative sample. The mode I and mode II fracture toughness of untoughened laminate as well as the 15 gsm fiber veils interleaved laminate were carried out at 200 and 250 °C by DCB and ENF measurements, respectively, and their corresponding fracture surface morphology was observed by SEM.

**Figure 9:**
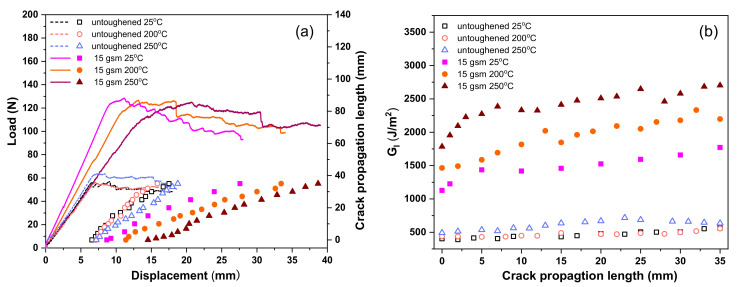
DCB test results of untoughened laminate and the 15 gsm fiber veils interleaved laminate measured at different temperatures: (**a**) Load–displacement curves and crack propagation length–displacement curves, as well as (**b**) the corresponding R-curves.

Figure 9 presents the load–displacement and crack propagation length–displacement curves of the untoughened laminate and the 15 gsm fiber veils interleaved laminate measured at 25, 200, and 250 °C, respectively. All load–displacement curves show a similar tendency, irrespective of the testing temperatures, which means the characteristic of interlaminar fracture behavior has not changed greatly at high temperatures. It is also found that the load–displacement curves for the untoughened laminate show a slight increase of maximum load force at the crack initiation stage with the temperature, while those for the 15 gsm fiber veils interleaved laminate exhibit a remarkable increase of displacement without changing the maximum load. It is suggested that the G_IC_ is enhanced at high temperatures for both laminates. Moreover, the load force in the fluctuation stage of the load–displacement curves for these laminates is raised with the enhancement of the testing temperature; meanwhile, the slope of the corresponding crack propagation length–displacement curve decreased, which means the G_IR_ values were also enhanced at a high temperature.

The effect of temperature on mode I interlaminar toughness of the untoughened and 15gsm fiber veils interleaved laminates is elucidated by R-curves. The curves depict that the G_I_ values at initiation stage (G_IC_) and at propagation stage (G_IR_) of untoughened laminate only give a slight increase with the temperature. However, the interleaved laminate exhibits significantly enhanced G_IC_ and G_IR_ values with the temperature increase. The G_IC_ and G_IR_ values of untoughened and interleaved laminates tested at different temperatures are summarized in Table 2. It is indicted that the untoughened laminate exhibits a slight increase in the mode I fracture toughness as measured at a relatively high temperature. In addition, the interleaved laminate reveals the extremely higher mode I fracture toughness compared to the untoughened one, even at 200 and 250 °C. For example, the untoughened laminate gave the G_IC_ and G_IR_ values of 525 and 699 J/m^2^ as tested at 250 °C, which are 26% and 16% enhanced compared to that of the specimen tested at room temperature. On the other hand, the interleaved laminate tested at 250 °C exhibits G_IC_ and G_IR_ values as high as 1599 and 2237 J/m^2^, which increased by 37% and 45%, respectively, as compared to the corresponding specimens tested at 25 °C. Furthermore, the G_IC_ and G_IR_ values for the interleaved laminate can reach 205% and 220% increments in comparison with the untoughened laminate at 250 °C. It can be deduced that the incorporation of TPPI fiber veils in the laminate interlayer is an effective way to improve the mode I fracture toughness of the polyimide composite under both room temperature and elevated temperatures.

**Figure 10:**
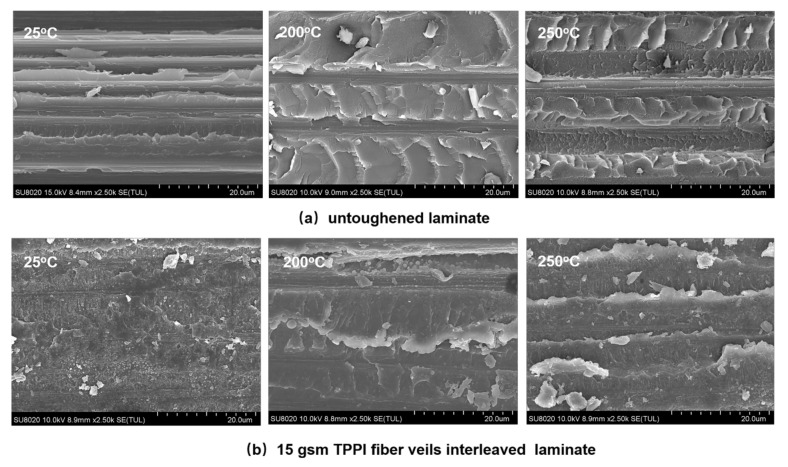
SEM micrographs of mode I fracture surfaces for untoughened and interleaved laminates after DCB test at different temperature. (**a**) untoughened laminate; (**b**) 15 gsm TPPI fiber veils interleaved laminate.

The SEM micrographs of fracture surfaces of the untoughened laminate and the 15 gsm fiber veils interleaved laminate after high-temperature DCB tests are shown in Figure 10. For the untoughened laminate, the flake-like deformed resin morphology can be observed for the specimen tested at elevated temperatures, instead of a flat and smooth surface morphology for that tested at room temperature. Moreover, the flake domains on the fracture surface increase with temperature, implying that the extent of matrix deformation is greatly increased at high temperatures. It can be deduced that the TSPI matrix resin exhibited an improved ductile behavior at an elevated temperature, which is considered to be the major reason for the higher mode I toughness. Similar results were confirmed by Czabaj et al. [44] and Boni et al. [45] with their experimental evidence.

For the 15 gsm fiber veils interleaved laminate, the morphology of the bicontinuous structure accompanied by the particulate phase can also be observed on the specimen surface after DCB test at 200 and 250 °C as mentioned above; consequently, the cracks would pass though the TPPI/TSPI matrix bicontinuous complex layer, leading to a cohesive failure. Moreover, there is a more distinctive shear lip structure (white banded patterns) detected by further fractographic examination for the 15 gsm fiber veils interleaved laminate tested at an elevated temperature. It is indicated that more apparent plastic deformation occurred in the interlayer of the specimen tested at a high temperature, leading to the improvement of the mode I toughness with the temperature [46].

**Figure 11:**
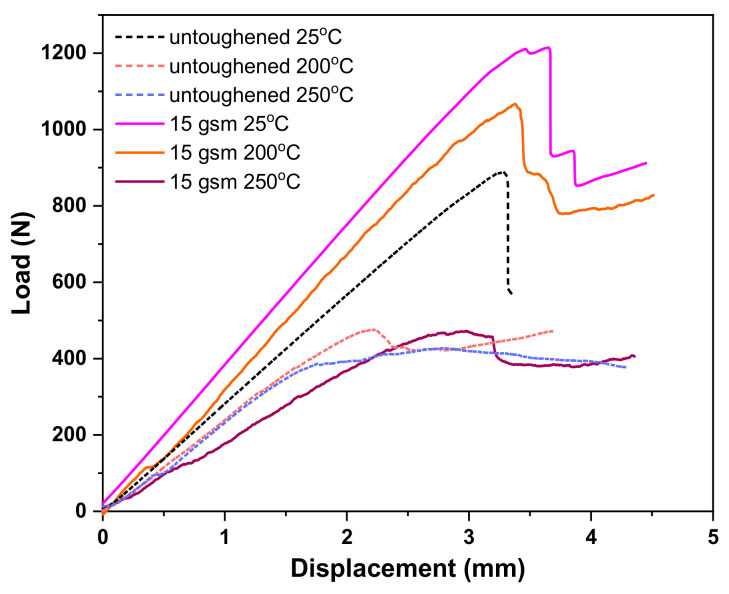
Load–displacement curves in ENF tests of untoughened and 15 gsm fiber veils interleaved laminates measured at different temperatures.

High-temperature ENF tests were performed to investigate the influence of high temperature on the mode II fracture toughness of the untoughened and interleaved laminates. Figure 11 shows the mode II load–displacement curves of the untoughened and the 15 gsm fiber veils interleaved laminate tested at different temperatures. It is noticed that the maximum load was severely reduced in high-temperature ENF tests for both the untoughened and interleaved laminates, which indicates a declining trend of their G_IIC_ property at a high temperature. However, the interleaved laminate always displays the better mode II toughness with much higher maximum load than the untoughened one, regardless of the temperature. According to the results summarized in Table 2, the G_IIC_ values for the 15 gsm fiber veils interleaved laminate are 1815 at 200 °C and 950 J/m^2^ at 250 °C, whereas those for the untoughened laminate are only 421 J/m^2^ at 200 °C and 258 J/m^2^ at 250 °C.

**Figure 12:**
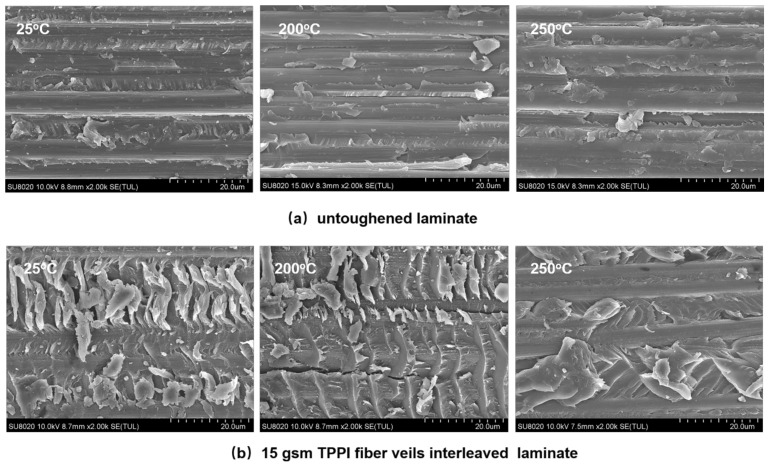
SEM micrographs of mode II fracture surfaces for untoughened and interleaved laminates after the ENF test at different temperature. (**a**) untoughened laminate; (**b**) 15 gsm TPPI fiber veils interleaved laminate.

The fracture surfaces of the untoughened and interleaved laminates after a high-temperature ENF test were recorded by SEM. As shown in Figure 12, it is found that the untoughened specimens after the ENF test at 200 and 250 °C still displays the fracture morphology of a relatively smooth surface with hackle-like resin fragments as that detected at room temperature. In addition, more microcracks induced by carbon fiber/matrix resin debonding clearly observed. This is the reason for the reduction in the G_IIC_ property as tested at a high temperature. It has been confirmed by Boni et al. [45] and Cowley et al. [47] that the degradation of the carbon fiber/matrix interfacial strength at a high temperature would lead to a decrease in the mode II fracture toughness. For the 15 gsm fiber veils interleaved laminate after the ENF test at 200 °C, the fracture surface shows the reduced banded structures due to the restrain of interlaminar crack crossing; meanwhile, the microcracks in the carbon fiber/matrix resin interface are detected. As the testing temperature further increases to 250 °C, the fracture surface of the interleaved laminate exhibits a further reduced banded structure in the fracture surface, and the carbon fibers are almost exposed because of the carbon fiber/matrix resin debonding to some extent. Therefore, the interfacial debonding is considered as the dominant failure mechanism for the interleaved composite at an elevated temperature, which leads to the decrease of G_IIC_ value. It is worth mentioning that the interleaved laminate still reveals a much rougher fracture surface caused by the existence of banded structures as compared with the untoughened one, despite the temperature. It can be concluded that the TPPI fiber veils interleaving is also effective to enhance the mode II fracture toughness of the CF/TSPI composite at an elevated temperature.

## 4. Conclusions

Thermally stable TPPI fiber veils with different areal densities were prepared by electrospinning and applied to interleave the CF/TSPI composite for improving the fracture toughness without sacrificing the heat resistance. All the TPPI fiber veils interleaved CF/TSPI laminates exhibit a simultaneous enhancement in the mode I and mode II fracture toughness as compared with the untoughened one. The phase structure in the interlayer of the interleaved laminates changes from a particulate phase to a bicontinuous TPPI and TSPI phase as the areal density of fiber veils increases from 5 to 15 gsm. The 15 gsm fiber veils interleaved laminate exhibits a significant improvement on fracture toughness with the maximum G_IC_ and G_IIC_ values increased by 179% and 132% as compared with the untoughened one. The morphology analysis for the mode I fracture toughness suggested that the crack deflection associated with the TPPI particles combined with the plastic deformation due to the TPPI component in the interlayer leads to the energy dissipation and absorption, which results in a remarkable increase in fracture toughness. According to the surface observation of the mode II fracture toughness, it is confirmed that the interleaved laminates reveal a banded structure, implying the energy dissipation is due to the interlaminar crack crossing. Moreover, the high-temperature DCB and ENF tests suggested that the TPPI fiber veils interleaved laminate also exhibits better fracture toughness than the untoughened one at an elevated temperature. The introduction of thermally stable TPPI fiber veils could enhance the mode I and mode II fracture toughness of CF/TSPI composites by exceeding 200% as compared to the untoughened one, even as tested at 250 °C. It can be concluded that interleaving the thermoplastic polyimide fiber veils into the polyimide composites is an efficient way to improve the toughness. Further investigation of the chemical structure of TPPI on the toughening effect and mechanism of polyimide composites should also be taken into consideration in the future research, as it will be helpful for the development of polyimide composites with high fracture toughness for advanced aviation and aerospace applications.

## Figures and Tables

**Table 1:** The results of DCB and ENF tests for the untoughened and interleaved laminates. The values in brackets represent the percentage increase of G_I_ and G_IIC_ values over the untoughened specimen.

Specimens	G_IC_ (J/m^2^)	G_IR_ (J/m^2^)	G_IIC_ (J/m^2^)
untoughened	418 ± 41	602 ± 51	881 ± 111
5 gsm	671 ± 76 (61%)	928 ± 33 (54%)	1079 ± 144 (22%)
10 gsm	1094 ± 133 (162%)	1334 ± 84 (122%)	1607 ± 189 (82%)
15 gsm	1167 ± 148 (179%)	1542 ± 105 (156%)	2042 ± 183 (132%)
20 gsm	733 ± 90 (75%)	1265 ± 26 (110%)	1803 ± 44 (105%)

**Table 2:** The results of DCB and ENF tests for the untoughened laminate and the 15 gsm fiber veils interleaved laminate measured at different temperatures. The values in brackets represent the percentage increase of G_I_ and G_IIC_ values over the untoughened specimen at the corresponding temperature.

Specimens	Test Temperature (°C)	G_IC_ (J/m^2^)	G_IR_ (J/m^2^)	G_IIC_ (J/m^2^)
untoughened	25	418 ± 41	602 ± 51	881 ± 111
	200	470 ± 30	640 ± 102	421 ± 21
	250	525 ± 25	699 ± 59	258 ± 21
15 gsm	25	1167 ± 148 (179%)	1542 ± 105 (156%)	2042 ± 183 (132%)
	200	1366 ± 92 (191%)	1832 ± 149 (186%)	1815 ± 109 (331%)
	250	1599 ± 133(205%)	2237 ± 188 (220%)	950 ± 84 (268%)

## Data Availability

The authors confirm that the data supporting the findings of this study is available within the article.
